# microRNA-125a-3p is regulated by MyD88 in *Legionella pneumophila* infection and targets NTAN1

**DOI:** 10.1371/journal.pone.0176204

**Published:** 2017-04-26

**Authors:** Elisa Jentho, Malena Bodden, Christine Schulz, Anna-Lena Jung, Kerstin Seidel, Bernd Schmeck, Wilhelm Bertrams

**Affiliations:** 1 Institute for Lung Research/iLung, German Center for Lung Research, Universities of Giessen and Marburg Lung Center, Philipps-University Marburg, Marburg, Germany; 2 Department of Medicine, Pulmonary and Critical Care Medicine, University Medical Center Marburg, Philipps-University Marburg, Marburg, Germany; University of Texas MD Anderson Cancer Center, UNITED STATES

## Abstract

**Background:**

*Legionella pneumophila* (*L*. *pneumophila*) is a causative agent of severe pneumonia. It is highly adapted to intracellular replication and manipulates host cell functions like vesicle trafficking and mRNA translation to its own advantage. However, it is still unknown to what extent microRNAs (miRNAs) are involved in the *Legionella*-host cell interaction.

**Methods:**

WT and *MyD88*^-/-^ murine bone marrow-derived macrophages (BMM) were infected with *L*. *pneumophila*, the transcriptome was analyzed by high throughput qPCR array (microRNAs) and conventional qPCR (mRNAs), and mRNA-miRNA interaction was validated by luciferase assays with 3´-UTR mutations and western blot.

**Results:**

*L*. *pneumophila* infection caused a pro-inflammatory reaction and significant miRNA changes in murine macrophages. In *MyD88*^-/-^ cells, induction of inflammatory markers, such as *Ccxl1/Kc*, *Il6* and miR-146a-5p was reduced. Induction of miR-125a-3p was completely abrogated in *MyD88*^-/-^ cells. Target prediction analyses revealed N-terminal asparagine amidase 1 (NTAN1), a factor from the n-end rule pathway, to be a putative target of miR-125a-3p. This interaction could be confirmed by luciferase assay and western blot.

**Conclusion:**

Taken together, we characterized the miRNA regulation in *L*. *pneumophila* infection with regard to MyD88 signaling and identified NTAN1 as a target of miR-125a-3p. This finding unravels a yet unknown feature of *Legionella*-host cell interaction, potentially relevant for new treatment options.

## Introduction

*Legionella pneumophila* (*L*. *pneumophila*), a gram-negative bacterium, is described as a causative pathogen of lung inflammation and life-threatening pneumonia [1]. Outbreaks still occur [2], and cases with as many as 450 patients have been reported [3]. Alveolar macrophages, which protect the lung from inhaled microorganisms and other insults, are their predominant host in the human lung. Upon infection, *L*. *pneumophila* form their replication niche inside macrophages, the *Legionella*-containing vacuole (LCV), where they replicate until host cell lysis. Infection of macrophages with *L*. *pneumophila* leads to a broad activation of signaling pathways, triggered by both extracellular and intracellular receptor molecules, such as Toll-like receptors (TLRs) that recognize pathogen associated molecular patterns (PAMPs). Upon formation of the LCV, *Legionella* shuttles an array of effector molecules into the host cytosol via type IV (T4S) and type II (T2S) secretion systems. The T2S –translocated effectors seem to attenuate MyD88 signaling and to enhance bacterial growth in mammalian host cells [4]. MyD88 is a central adaptor molecule that links all TLRs except TLR3 to their downstream signaling cascades. The consequence of signal cascade activation is typically the activation of gene transcription events that in sum constitute an anti-microbial defense. Coding and non-coding RNAs are both part of this response. microRNAs (miRNAs) are an example of short non-coding RNAs which are involved in the defense mechanisms of the host. Exemplarily, miR-146a-5p is broadly described as a negative regulator of IRAK1 and TRAF6, thereby limiting the immune response in a negative feedback loop [5]. By subduing the MyD88 response, *Legionella* is actively interfering with these processes. Therefore, it is still unknown to what extent miRNAs are involved in *L*. *pneumophila* infection, either as part of the host response, or as part of host cell rewiring by the pathogen. We characterized miRNA expression in murine macrophages upon *L*. *pneumophila* infection in the context of a *MyD88* knockout to shed more light on this particular aspect of host-pathogen interaction. We found significant changes of miRNA expression, which partly depended on the MyD88-pathway. Specifically, miR-125a-3p was found to be regulated in a MyD88-dependent manner. We could furthermore show that it targets NTAN1, an amidase which converts residual asparagine to aspartic acid, the first step in a series of protein modifications leading to eventual proteolysis. Regulation of NTAN1 by miR-125a-3p might be an important feature of protein stability control during *L*. *pneumophila* infection.

## Materials & methods

### Bacterial strains and infection

*L*. *pneumophila* Corby wild type was kindly provided by the Robert Koch Institute Berlin, Germany (A. Flieger, K. Heuner), and routinely grown as described previously [6]. This strain (Corby) has been used to establish the GenBank entry under accession number CP000675. Cells were infected with *L*. *pneumophila* at indicated multiplicity of infection (MOI).

### Cell culture and genotyping

The Raw264.7 cell line was obtained from the America Type Culture Collection (ATCC). Mouse embryonic fibroblasts (MEF) were kindly supplied by Bastian Stielow, Marburg, Germany. Both cell lines were cultured in DMEM medium with 10% FCS (PAA Laboratories, Pasching, Austria) without antibiotics. Raw264.7 cells were used from passage 2–15. MEF cells, used as a vehicle for luciferase reporter assay, were cultured from passage 53 to 60. Bone marrow-derived macrophages (BMMs) were freshly prepared from femurs and tibiae of C57BL/6 wild type (Charles River Laboratories, Wilmington, USA) and *MyD88*^*-/-*^ mice (kindly provided by C. Brunner, Ulm, Germany, [7]) and cultivated as described previously [8]. All cells were authenticated by microscopic morphology. Genotyping PCR for the *MyD88*^*-/-*^ cells was performed using primers for the *MyD88* gene (NC_000075.6) 5´-AGACAGGCTGAGTGCAAACTTGGTCTG-3´ (Primer A) and 5´-AGCCTCTACACCCTTCTCTTCTCCACA-3´ (Primer B) and with a primer for the neomycin resistance cassette, 5´-ATCGCCTTCTATCGCCTTCTTGACGAG-3´ (Primer C) (S1 Fig).

### Quantitative RT-PCR

RNA was isolated using Isol-RNA Lysis Reagent (5 PRIME, Hamburg, Germany), quantified by Nanodrop and reverse transcribed with the High Capacity cDNA Reverse Transcription Kit or the microRNA reverse transcription kit (both Life Technologies) according to the manufacturer’s protocol. Quantitative RT-PCR was performed on a ViiA™ 7 Real-Time PCR System using Fast SYBR Green or Taqman Fast Advanced Master Mix (both Life Technologies). Primer sequences were obtained from the PrimerBank database (https://pga.mgh.harvard.edu/primerbank/) or from the referenced publications. The following primers were used: *Ntan1* (NM_010946, sense: 5´-GGCATCGCTGTCAACATTAAAAC-3´, antisense: 5´-AATGCTAATCATTGGGCCTCC-3´, PrimerBank ID 87299633c2), *Cxcl1/Kc* (NM_008176, sense: 5´-ACTGCACCCAAACCGAAGTC-3´, antisense: 5´- TGGGGACACCTTTTAGCATCTT-3´, PrimerBank ID 229577225c1), *Il6* (NM_031168, sense: 5´- CTGCAAGAGACTTCCATCCAG-3´, antisense: 5´-AGTGGTATAGACAGGTCTGTTGG-3´, PrimerBank ID 13624310c1), *Sesn1* (NM_001162908, sense: 5´-ACACGGGATGCATGTCCCAAC-3´, antisense: 5´- TCCCACATCTGGATAGAGACGATTCA-3´, [9]), *Gapdh* (NM_001289726.1, sense: 5´-TGATGGGTGTGAACCACGAG-3´; antisense: 5´-TCAGTGTAGCCCAAGATGCC-3´, [10]). Commercial microRNA primers were purchased from Life Technologies. snoRNA202 served as endogenous control for microRNA analyses, while *Gapdh* was used for mRNA analyses. Samples were run in technical triplicates. Data were processed with the ViiA7 software (V. 1.2.4., Life Technologies) and analyzed with the 2^-ddCt^ method [11].

### High troughput qPCR and data analysis

Taqman Low Density Arrays (TLDAs) were performed with RNA samples from infected and uninfected WT and *MyD88*^*-/-*^ BMMs according to the manufacturer´s instructions. For data interpretation, the Bioconductor R package HTqPCR (version 3.2) [12] with the limma package (version 3.24.15) [13] was used. The U6 snRNA was used for normalization.

### Cloning

The 3´UTR of *Ntan1* was amplified from murine macrophage cDNA with the following custom made primers: sense: 5´-GTTTTCTCGAGggagctaacatcagctcagag-3´; antisense 5´- GTTTTGCGGCCGCgaaagacaggaaggacggaag-3´. The 5´overhang (GTTTT) and restriction sites for XhoI (CTCGAG) and NotI (GCGGCCGC) are shown in capital letters. The amplified fragment was inserted into the psiCheck2 vector (Promega). The mutated 3´UTR of *Ntan1* was purchased as a custom made gBlocks gene fragment from Integrated DNA Technologies.

### Transfection

For the Luciferase Reporter Assay, mouse embryonic fibroblasts (MEF) were co-transfected with (1) synthetic miR-125a-3p or a chemistry-matched scramble sequence at a final concentration of 50 nM and (2) 500 ng of psiCheck2 vector (Promega) containing either the wild type or mutated 3´UTR of *Ntan1*. For western blot analysis of NTAN1 protein abundance, Raw264.7 cells were transfected with synthetic miR-125a-3p or a chemistry-matched scramble sequence at a final concentration of 5 nM. Transfections were carried out with siPort NeoFX (Ambion) for Raw264.7 cells or Lipofectamine 2000 (Thermo Fisher) for MEF cells, according to the manufacturers´ recommendations.

### Western blot

Western blot was performed as described previously [14]. Immunodetection was carried out with anti-NTAN1 (antikoerper-online.de, ABIN967387) and anti-tubulin (Santa Cruz, sc-5286) and visualized on a chemoluminescence imager (INTAS Science Imaging Instruments, Göttingen, Germany).

### ELISA

ELISA against KC was performed with the Mouse CXCL1/KC DuoSet ELISA (R&D Systems) according to the manufacturer´s recommendations.

### Luciferase reporter assay

At 72 h after transfection, MEF cells were lysed and substrates for Renilla and Firefly Luciferase were added, using the Dual Glo Assay System according to the manufacturer´s recommendations (Promega). Luminescence emission was detected on a Tecan Infinite M200 Pro.

### Ethical statement

Animals were handled according to the EU council directive 86/609/EEC for the protection of animals and sacrificed by intraperitoneal administration of a fatal dose of ketamine and xylazine. The performed protocols were approved by the responsible animal ethics committee (Philipps-University Marburg; permit number: EX-22-2013), and all efforts were made to minimize suffering.

### Statistical analyses

Data are presented as mean & SD (standard deviation) of at least three independent experiments. Effects were statistically evaluated employing the indicated appropriate tests. p-values < 0.05 were considered significant and adjusted for multiple testing where indicated.

## Results

### *L*. *pneumophila* characteristically changes expression of key miRNAs and mRNAs in a MyD88 dependent manner

**Fig 1:**
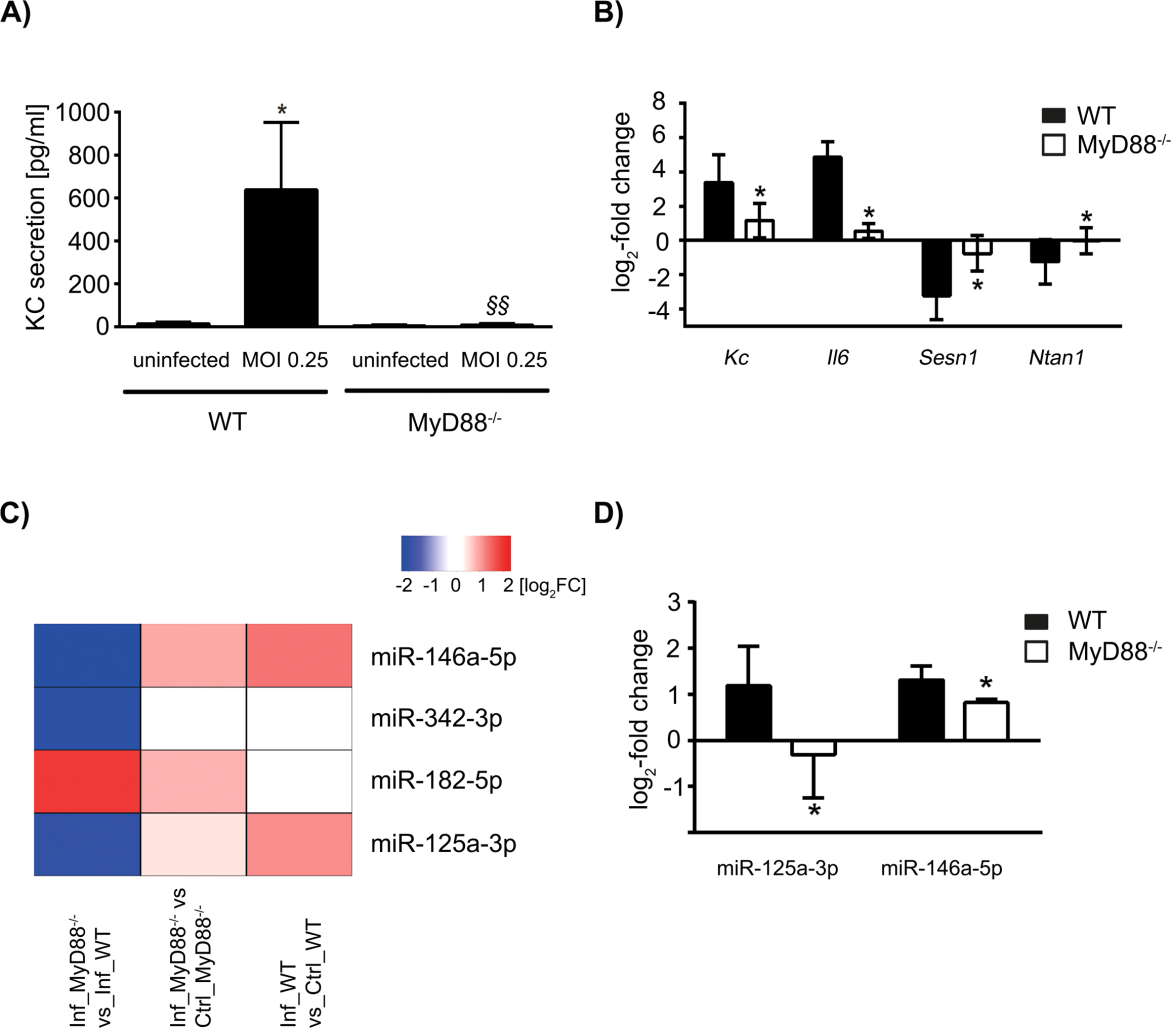
KC, IL-6 and microRNAs are differentially regulated in wild type and *MyD88*^*-/-*^ BMMs upon infection with *Legionella pneumophila*. WT and *MyD88*^*-/-*^ macrophages were infected with *L*. *pneumophila* for 24 h at a MOI of 0.25, and KC release was determined by ELISA (A). The expression patterns of selected mRNAs were analysed by qPCR (B). microRNAs were investigated by Taqman Low Density Array. The top 4 differentially regulated microRNAs as ranked by p-value are shown. The relative log_2_ fold induction in infected *MyD88*^*-/-*^ vs. infected WT cells (left column), in infected *MyD88*^*-/-*^ vs. uninfected *MyD88*^*-/-*^ *cells* (middle column) or in infected WT vs. uninfected WT cells (right column) is depicted (C). Selected microRNAs were validated by qPCR (D). mRNA samples were normalized against GAPDH, while microRNA samples were normalized against snRNA U6 (C) or snoRNA202 (D). Data are shown as mean & SD of at least three independent experiments. Statistical tests were one-way ANOVA with post-hoc intergroup comparison (A) and Student´s T-Test with Bonferroni-Holm adjustment for multiple testing (B) and (D). *p<0.05 WT uninfected vs. WT MOI 0.25, ^§§^p<0.05 *MyD88*^*-/-*^ MOI 0.25 vs. WT MOI 0.25 (A), *p_adj_<0.05 *MyD88*^*-/-*^ vs. WT (B) and (D).

Firstly, we aimed to establish a solid set of markers to ensure efficient infection of BMMs with *L*. *pneumophila* and to show the perturbations inflicted by the *MyD88* knockout. We detected increased expression of pro-inflammatory KC (protein and mRNA) and *Il6* (mRNA) in WT BMMs and to a significantly lesser extent also in *MyD88*^-/-^ cells at 24 h post infection (p.i., Fig 1A and 1B). Additionally, we confirmed a reduction in *Sesn1* mRNA levels upon infection as previously described [9], which was significantly stronger in WT BMMs than in *MyD88*^-/-^ BMMs at 24 h p.i. These established markers substantiated the pro-inflammatory macrophage phenotype that was expected to manifest after infection. In addition, we show *Ntan1*, which was down-regulated in WT cells, but was not regulated in *MyD88*^*-/-*^ BMMs upon infection (Fig 1B).

We furthermore characterized the response of WT and *MyD88*^-/-^ BMMs on the miRNA level by Taqman Low Density Array. We found the miRNAs miR-146a-5p, miR-342-3p, miR-182-5p and miR-125a-3p to be subject to significant (p<0.05) differential expression between WT and *MyD88*^-/-^ BMMs (Fig 1C). Within the scope of this work, we chose two miRNAs for a more detailed analysis on the basis of their immunomodulatory potential. In three additional independent confirmatory experiments, the well-characterized miR-146a-5p was up-regulated 24 h p.i. in WT cells to a significant extent when compared to *MyD88*^-/-^ BMMs. Furthermore, miR-125a-3p was induced 24 h post *L*. *pneumophila* infection in WT BMMs but conversely showed a down-regulation in *MyD88*^*-/-*^ BMMs (Fig 1D). This microRNA has been described to play a role in autophagy [15], obesity [16] and cancer [17, 18]. By *in silico* analysis (TargetScan Mouse Release 7.1), we found that binding site topology in the 3’ UTR of *Ntan1* strongly suggests an interaction with miR-125a-3p. We subsequently investigated the relationship between miR-125a-3p and *Ntan1*, which both seem to be subject to downstream MyD88 signaling.

### miR-125a-3p functionally binds the *Ntan1* 3´-UTR and regulates NTAN1 protein abundance

**Fig 2:**
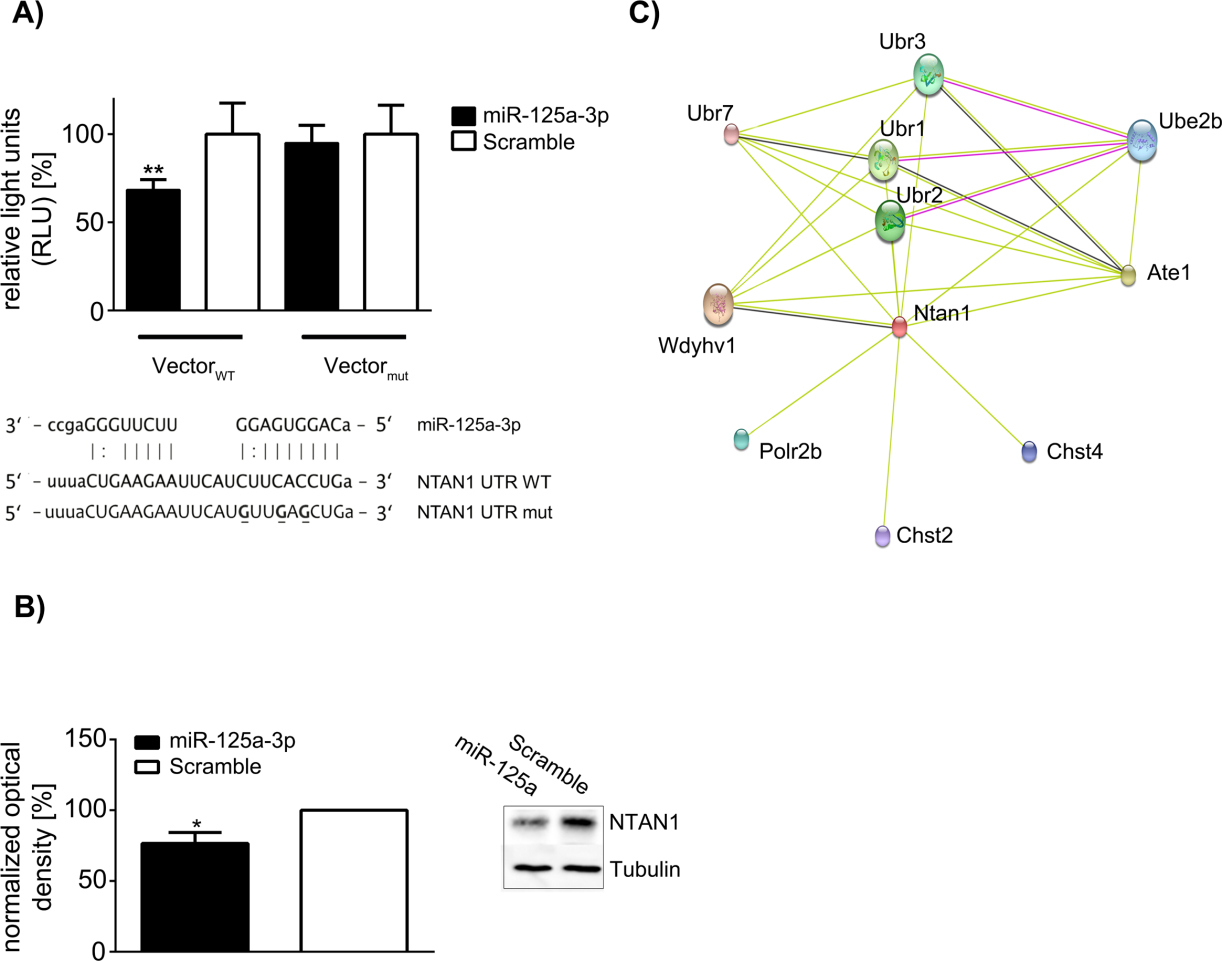
microRNA-125a-3p targets NTAN1 and regulates it on protein level. microRNA-125a-3p shows partial homology to the 3´UTR of *Ntan1* mRNA. To destroy the sequence compatibility in the seed region, 3 bases were exchanged (bold, underlined). MEF cells were co-transfected with the microRNA-125a-3p or a scramble sequence and the psiCheck2 vector carrying either the wild type *Ntan1* 3´UTR or the mutated sequence. Relative luminescence units (RLU) were determined after 72 h. Renilla luciferase signal was normalized against firefly luciferase. Relative luminescence units were calculated as a percentage of signal in scramble transfected cells (A). Raw264.7 cells were transfected with microRNA-125a-3p or a scramble sequence (5 nM), and NTAN1 protein levels were determined by western blot after 72 h. Densitometric analysis was performed with normalization against Tubulin. One representative blot is shown (B). In an interaction network, which was built from the STRING database, NTAN1 is shown to be associated with the N-end rule pathway. The observed interactions are based on experimental data (purple edges), co-expression (black edges) and textmining (green edges). Only direct interactions with an interaction score of minimum 0.4 (i.e. medium confidence) are shown. Interaction partners include arginyltransferase 1 (Ate1) and members of the ubiquitin protein ligase E3 component n-recognin (Ubr) family (C). Data are shown as mean & SD of at least four independent experiments. Statistical tests were one-way ANOVA with post-hoc intergroup comparison (A) and Mann Whitney U test (B). **p<0.01 Vector WT + miR-125a-3p vs. Vector Mutant + miR-125a-3p (A), *p<0.05 miR-125a-3p vs. Scramble (B).

The hypothetical binding site for miR-125a-3p is partly conserved among several species, which indicates selective pressure on this site and therefore suggests a conserved function. We constructed a luciferase reporter vector including the 3’ UTR of *Ntan1* to validate this putative molecular interaction. miR-125a-3p was capable of significantly decreasing the luminescence signal by approx. 30% when compared to a scramble control RNA. The mutation of 3 bases within the 3´UTR abrogated this effect (Fig 2A). Having found that this miRNA had no effect on *Ntan1* transcript levels, we performed western blot and observed that overexpression of miR-125a-3p reduced NTAN1 protein by approx. 23% as opposed to scramble transfected control cells (Fig 2B). Furthermore, a network analysis based on the STRING database [19] highlights the involvement of NTAN1 in the n-end rule pathway by association of NTAN1 with the ubiquitin protein ligase E3 component n-recognin (Ubr) family and arginyltransferase 1 (Ate1, Fig 2C). In conclusion, we herewith reveal an interaction of the *L*. *pneumophila*-induced miR-125a-3p with NTAN1.

## Discussion

The aim of this study was to find miRNA-regulated events during *L*. *pneumophila* infection in dependency of the central signal transduction molecule MyD88. miRNAs constitute a cellular transcript control mechanism which is instrumental to the immune response but also constitutes a potential target for host cell subversion by a pathogen [20]. We infected murine WT and *MyD88*^*-/-*^ BMMs to help unravel this particular aspect of host-pathogen interaction.

*L*. *pneumophila* is an intracellular pathogen that causes shifts in the gene expression of the host cell upon infection. While the cellular immune response aims to remove the pathogen, *L*. *pneumophila* manipulates host gene expression to its own advantage [21]. We have described previously that TNFAIP2 is up-regulated upon *L*. *pneumophila* infection, a factor which is beneficial for bacterial replication inside the cell [22]. Aside from coding RNAs, non-coding RNAs such as miRNAs are also regulated upon infection with *L*. *pneumophila*. In a high throughput screening by Taqman Low Density Array, we identified, among others, miR-146a-5p and miR-125a-3p as significantly regulated between WT and *MyD88*^*-/-*^ BMMs. Subsequent further testing confirmed regulation of miR-146a-5p, which has been extensively characterized with regard to its anti-inflammatory properties [23], in WT and *MyD88*^*-/-*^ BMMs. While markedly weaker in *MyD88*^*-/-*^ BMMs, this miRNA was induced in both cases. This suggests that MyD88 is not strictly required but merely an accessory to the induction of miR-146a-5p. In accordance with this finding, pro-inflammatory cytokines showed a lower induction on mRNA level in *MyD88*^*-/-*^ cells when compared to WT cells, and KC secretion was close to the detection limit (Fig 1A). The induction of miR-125a-3p, however, revealed a strong dependence on MyD88, as it was down-regulated in *MyD88*^*-/-*^ cells upon infection, while robustly induced in WT cells. The induction of this miRNA under infectious conditions and its dependency on TLR2 and MyD88 has been shown previously [24]. We found a functional interaction between miR-125a-3p and the *Ntan1* 3´UTR by Luciferase Reporter Assay, which was abolished by the mutation of three bases in the miRNA-binding region of the *Ntan1* 3´UTR. Whilst no detectable down-regulation of *Ntan1* transcript was achieved upon artificial overexpression of miR-125a-3p, we confirmed down-regulation on the protein level by western blot.

NTAN1 is an aminohydrolase which converts N-terminal asparagine to aspartic acid within the N-end rule pathway of protein degradation [25]. While its involvement in *Legionella* infection remains to be elucidated, the N-end rule pathway is known to play a role in antigen degradation and the generation of epitopes during infection with *Listeria monocytogenes* [26, 27], a pathogen also known to induce miR-125a-3p [24].

Our study reveals the NTAN1/miR-125a-3p interaction as a new feature of the host´s response against *L*. *pneumophila*. Intriguingly, *Legionella* have developed sophisticated mechanisms of host cell exploitation during their co-evolution with eukaryotic host cells. They secrete more than a hundred effector proteins into the host cytosol [28], and have been described to hijack the host proteasome by specifically labelling factors with ubiquitin for degradation [29]. NTAN1 as part of the protein degradation machinery is associated with several E3 ubiquitin protein ligases (Fig 2C). MyD88-dependent regulation of miR-125a-3p may prove to be a mechanism to control antigen stability and epitope generation via NTAN1 and the N-end rule pathway upon bacterial infection.

The integration of these events into one regulatory unit is supported by the fact that another member of the miR-125 family, miR-125b-5p, has been shown to target MyD88 [30]. In our high-throughput dataset, while not statistically significant, this microRNA shows an up-regulation in the wild type upon infection (1.26 log_2_ FC) and a down-regulation in *MyD88*^*-/-*^ cells (-2.06 log_2_ FC), mirroring the expression pattern we show for miR-125a-3p (Fig 1D). We suggest the existence of a functional, self-regulating node in infection, which contains MyD88, miR-125a-3p, miR-125b-5p and NTAN1. It remains to be studied whether this mechanism works in favor of *Legionella*, or whether it antagonizes it. In addition, our observations are based on primary murine cells and will have to be validated in human cells.

In summary, this study characterizes for the first time changes in miRNA expression upon macrophage infection by *L*. *pneumopila* in the context of *MyD88* deletion. We describe the regulation of the factor NTAN1 by miR-125a-3p, which is MyD88-dependent. This finding underlines the importance of miRNAs in innate immune reactions against bacterial pathogens and adds understanding to a node downstream of MyD88 in the complex interaction network between bacterium and host cell [28].

## Supporting information

S1 Fig Genotype validation of the used BMMs. PCR was performed to validate the WT and *MyD88*^*-/-*^ genotype of the used BMMs. The primer pair for the *MyD88* gene (A+B) yielded a product only in the WT cells, while the primer pair designed to detect the inserted neomycin resistance cassette (B+C) only yielded a product in the *MyD88*^*-/-*^ cells. A no-template control (NTC) was included to ensure method fidelity.
(TIF)
