# Supplementary figures and images for: microRNA-125a-3p is regulated by MyD88 in Legionella pneumophila infection and targets NTAN1
Source: PLoS One. 2017 Apr 26;12(4):e0176204. doi: 10.1371/journal.pone.0176204 (PMC5406027; doi:10.1371/journal.pone.0176204)

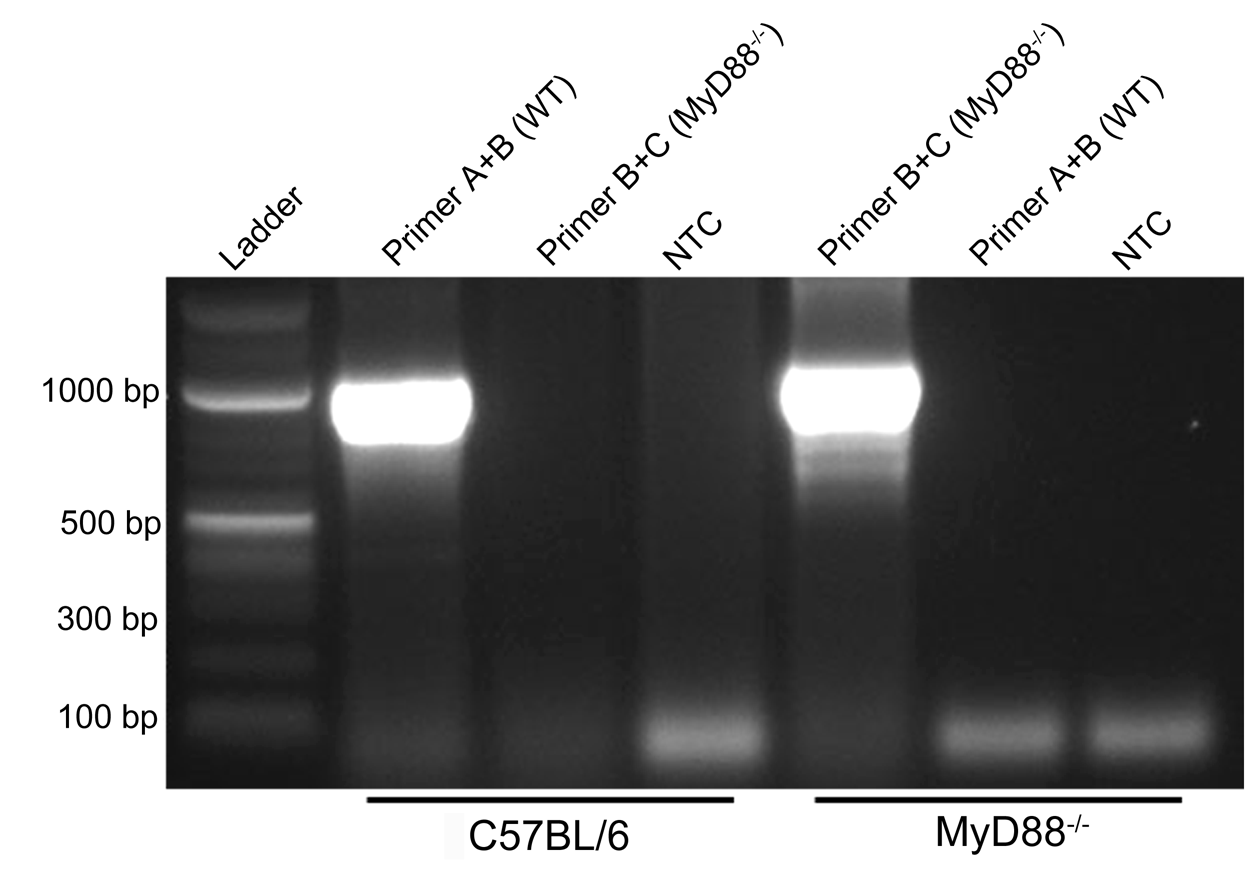

Supplement: S1 Fig — PCR was performed to validate the WT and MyD88-/- genotype of the used BMMs. The primer pair for the MyD88 gene (A+B) yielded a product only in the WT cells, while the primer pair designed to detect the inserted neomycin resistance cassette (B+C) only yielded a product in the MyD88-/- cells. A no-template control (NTC) was included to ensure method fidelity. (TIF) [file pone.0176204.s001.tif]
